# Noncoding RNA blockade of autophagy is therapeutic in medullary thyroid cancer

**DOI:** 10.1002/cam4.355

**Published:** 2014-12-08

**Authors:** Justin S Gundara, JingTing Zhao, Anthony J Gill, James C Lee, Leigh Delbridge, Bruce G Robinson, Catriona McLean, Jonathan Serpell, Stan B Sidhu

**Affiliations:** 1 Cancer Genetics, Kolling Institute of Medical ResearchSt. Leonards, Sydney, New South Wales, Australia; 2 Endocrine Surgical Unit, Royal North Shore HospitalSt. Leonards, Sydney, New South Wales, Australia; 3 Department of Anatomical Pathology, Royal North Shore HospitalSt. Leonards, Sydney, New South Wales, Australia; 4 Department of Endocrinology, Royal North Shore Hospital, University of SydneySt. Leonards, Sydney, New South Wales, 2065, Australia; 5 Department of Anatomical Pathology, Alfred Hospital, Monash UniversityPrahran, Melbourne, Victoria, Australia; 6 Endocrine Surgical Unit, Alfred Hospital, Monash UniversityPrahran, Melbourne, Victoria, 3181, Australia

**Keywords:** Autophagamir, autophagy, medullary, miR-9-3p, MTC, oncophagy

## Abstract

Micro-RNAs are dysregulated in medullary thyroid carcinoma (MTC) and preliminary studies have shown that miRNAs may enact a therapeutic effect through changes in autophagic flux. Our aim was to study the in vitro effect of miR-9-3p on MTC cell viability, autophagy and to investigate the mRNA autophagy gene profile of sporadic versus hereditary MTC. The therapeutic role of miR-9-3p was investigated in vitro using human MTC cell lines (TT and MZ-CRC-1 cells), cell viability assays, and functional mechanism studies with a focus on cell cycle, apoptosis, and autophagy. Post-miR-9-3p transfection mRNA profiling of cell lines was performed using a customized, quantitative RT-PCR gene array card. This card was also run on clinical tumor samples (sporadic: *n* = 6; hereditary: *n* = 6) and correlated with clinical data. Mir-9-3p transfection resulted in reduced in vitro cell viability; an effect mediated through autophagy inhibition. This was accompanied by evidence of G2 arrest in the TT cell line and increased apoptosis in both cell lines. Atg5 was validated as a predicted miR-9-3p mRNA target in TT cells. Post-miR-9-3p transfection array studies showed a significant global decline in autophagy gene expression (most notably in *PIK3C3*, *mTOR*, and *LAMP-1*). Autophagy gene mRNAs were generally overexpressed in sporadic (vs. hereditary MTC) and *Beclin-1* overexpression was shown to correlate with residual disease. Autophagy is a tumor cell survival mechanism in MTC that when disabled, is of therapeutic advantage. *Beclin-1* expression may be a useful prognostic biomarker of aggressive disease.

## Introduction

Medullary thyroid cancer (MTC) accounts for 3–10% of all thyroid malignancies and can be classed as “sporadic” (SMTC; 75%) or “hereditary” (HMTC; 25%) on the basis of germline *RET* gene mutation status and clinical phenotype 1. Discovery of the *RET* gene mutation within the context of MTC 20 years ago forced a paradigm shift in the way in which clinicians now manage this disease 2. Beyond *RET*, however, there remains a void in our understanding of the molecular biology of SMTC.

Given significant biomarker and therapeutic potential, noncoding RNAs have been the focus of major research efforts 3,4. More specifically, micro-RNAs (miRNAs) are small, non-protein-coding nucleotides (18–25 bp) that negatively regulate messenger RNA (mRNA), and thus, posttranscriptional protein expression 5,6. Recent studies have proven miRNAs to be of fundamental importance throughout embryological development, tissue differentiation, proliferation, and cell death 5.

Our laboratory has previously reported significant differential miRNA expression between SMTC and HMTC 7. MiR-9-3p was shown to be underexpressed in sporadic cases and functional work showed that miRNA therapeutics were efficacious in vitro. MiR-9-3p is one member of the miR-9 family and has been shown to be dysregulated in not only MTC but also breast cancer cell lines where it has also been proven to synergize with MEK inhibitors to suppress cell growth 8. Roles in differentiation of pluripotent osteoblastic stem cells have also been published 9 but beyond these present findings, there is a relative paucity of data upon which to reflect regarding the precise role that miR-9-3p plays in biology.

The suggestion of a therapeutic mechanism based on miRNA regulation of autophagy (a ubiquitous process of cellular subsistence) has also been raised in our prior studies, and this is a theme that has subsequently gained further research interest under the banner of “oncophagy” 10.

Despite the innumerable and significant molecular advances that have occurred over the last three decades, improved survival outcomes for patients with MTC (and sporadic disease in particular) have not occurred 11,12. Newer diagnostic, prognostic, and therapeutic strategies require development based on the burgeoning potential of noncoding RNA discovery. We aimed to explore the in vitro therapeutic impact of miR-9-3p in MTC with an emphasis on autophagy manipulation and to probe clinical correlations with key autophagy regulators.

## Materials and Methods

Ethical approval for all studies was granted by the Human Research Ethics Committees of the Northern Sydney Central Coast Area Health Service, the Alfred Hospital and the University of Sydney. Methods are detailed in Data S1.

## Results

### miR-9-3p transfection reduces TT and MZ-CRC cell viability

MTS assays performed at 48 h post transfection of pre-miR-9-3p or negative control demonstrated a significant impact on cell viability in both TT and MZ-CRC-1 cell lines. Mean TT cell viability (vs. negative control) was 77% (*P *<* *0.01) and in MZ-CRC-1 90% cell viability was evident (*P *=* *0.03). When comparing cell lines, the pre-miR-9-3p effect was significantly greater in the TT cell line (*P *<* *0.01).

**Figure 1:**
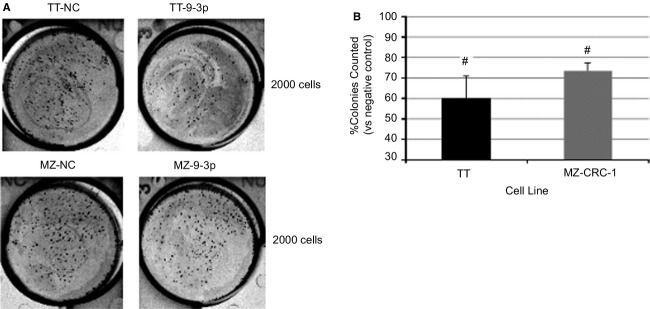
(A) Photo of stained TT and MZ-CRC-1 cell colonies 28 days post plating (representative of one experiment); (B) quantified numbers of cell colonies (expressed as a % of negative control-transfected cells; #*P *<* *0.05).

Additional clonogenic assays were performed to further explore the pre-miR-9-3p effect on cell viability. Figure1A and B demonstrates reduced colony counts in both TT and MZ-CRC-1 cells 28 days after plating (as compared with negative control transfected cells). TT cell colonies were reduced by 40% (*P *=* *0.018), whereas MZ-CRC-1 cell colonies were reduced by 26% (*P *=* *0.016).

### miR-9-3p transfection has a cell-line-dependent effect on cell cycle progression

After demonstration of an effect upon cell proliferation, the mechanism of this effect was explored. Figure S1 shows cell cycle analysis results for TT (Fig. S1A) and MZ-CRC-1 (Fig. S1B) cell lines comparing negative control-transfected cells with miR-9-3p. TT cells demonstrated a significant effect on cell cycle progression, with miR-9-3p-transfected cells showing significantly lower G1 (*P *<* *0.01) and higher G2 (*P *<* *0.01) cell phase counts when compared to negative control. More specifically, the G1 cell population was reduced by 4.67% and the G2 population increased by 4.12%. This effect was not apparent in the MZ cell line, however. Of note was the absence of a significant increase in the sub-G1 population of cells in both cell lines following miR-9-3p transfection.

### miR-9-3p transfection inhibits autophagic flux and increases apoptosis

Prior to examining the impact of miRNAs upon autophagy and apoptosis pathways specifically, pharmacological induction (Rapamycin) and blockade (3-methyladenine; 3-MA) of autophagy were imposed. Figure S2A and B illustrates significantly increased cleaved poly (ADP-ribose) polymerase (cPARP) western blot expression following TT-cell treatment with 3-MA when compared to rapamycin (*P *=* *0.02). In addition, this figure demonstrates significant increases in autophagy markers, Atg5 and LC3BII (*P *<* *0.01) following pharmacological induction with rapamycin (compared to 3-MA blockade). These results served as controls for post-miR-9-3p transfection immunoblots on both cell lines.

**Figure 2:**
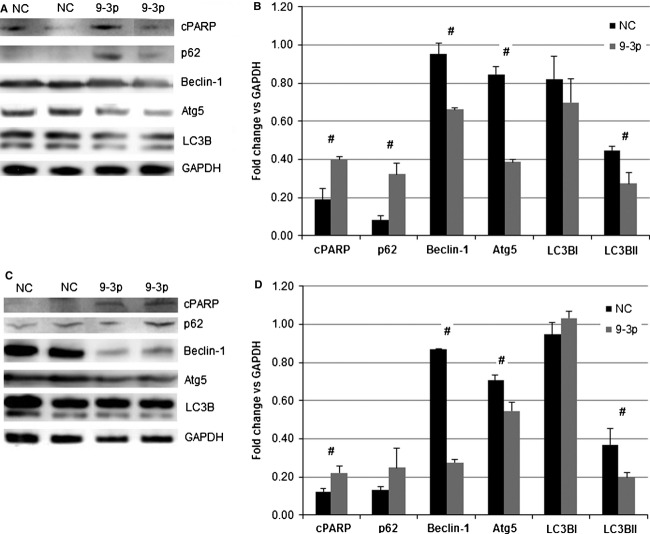
(A) Western blot of apoptosis and autophagy markers 48 h post-TT cell transfection with miR-9-3p or negative control; (B) quantified densitometry results of (A); (C) Western blot of apoptosis and autophagy markers 48 h post-MZ-CRC-1 cell transfection with miR-9-3p or negative control; (D) quantified densitometry results of C (#*P *<* *0.05).

Subsequent miR-9-3p transfected lysates from both cell lines resulted in evidence of a significant reduction in markers of autophagic flux (TT: Figure2A and B and MZ-CRC-1: C and D). Global changes in autophagy markers following miR-9-3p transfection were evident for all autophagy markers in both cell lines, with the exception of p62 in the MZ-CRC-1 cell line.

A concurrent and significant increase in cPARP expression was also seen in both cell lines, indicating enhanced apoptosis within the context of miR-9-3p-induced autophagic blockade.

### miR-9-3p transfection inhibits a range of autophagy-related genes

In order to explore miR-9-3p regulation of autophagy, a posttransfection autophagy gene mRNA array was performed. Following correction for false discovery, several autophagy genes were found to be significantly downregulated at the *P *<* *0.1 level (Table S2). Of note was the lack of suppressed gene crossover between cell lines.

**Figure 3:**
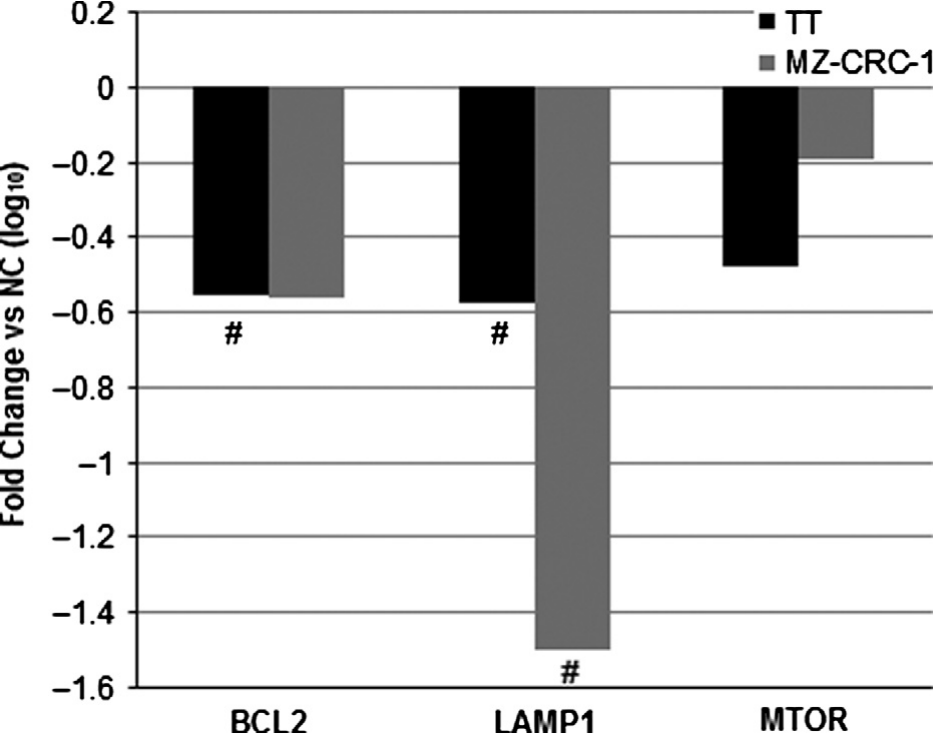
qPCR results displaying TT and MZ-CRC-1 expression of selected probes 48 h following miR-9-3p transfection (expressed as log_10_ fold change relative to control; #*P *<* *0.05).

Following array studies, *BCL2*, *LAMP1*, and *MTOR* were chosen as gene targets for qPCR validation. Figure3 illustrates a suggestion of downregulation of all three gene targets following miR-9-3p transfection in both cell lines when compared to negative control-transfected cells (expressed as log_10_ fold change). More specifically, *BCL2* was suppressed in TT cells significantly (*P *=* *0.014) but not MZ-CRC-1 cells (*P *=* *0.083). *LAMP1* was downregulated in both cell lines significantly (TT: *P *=* *0.004; MZ-CRC-1: *P *=* *0.025) and *MTOR* was not significantly suppressed in either cell line (TT: *P *=* *0.154; MZ-CRC-1: *P *=* *0.079).

### miR-9-3p transfection results in cell-line-specific Atg5 inhibition

**Figure 4:**
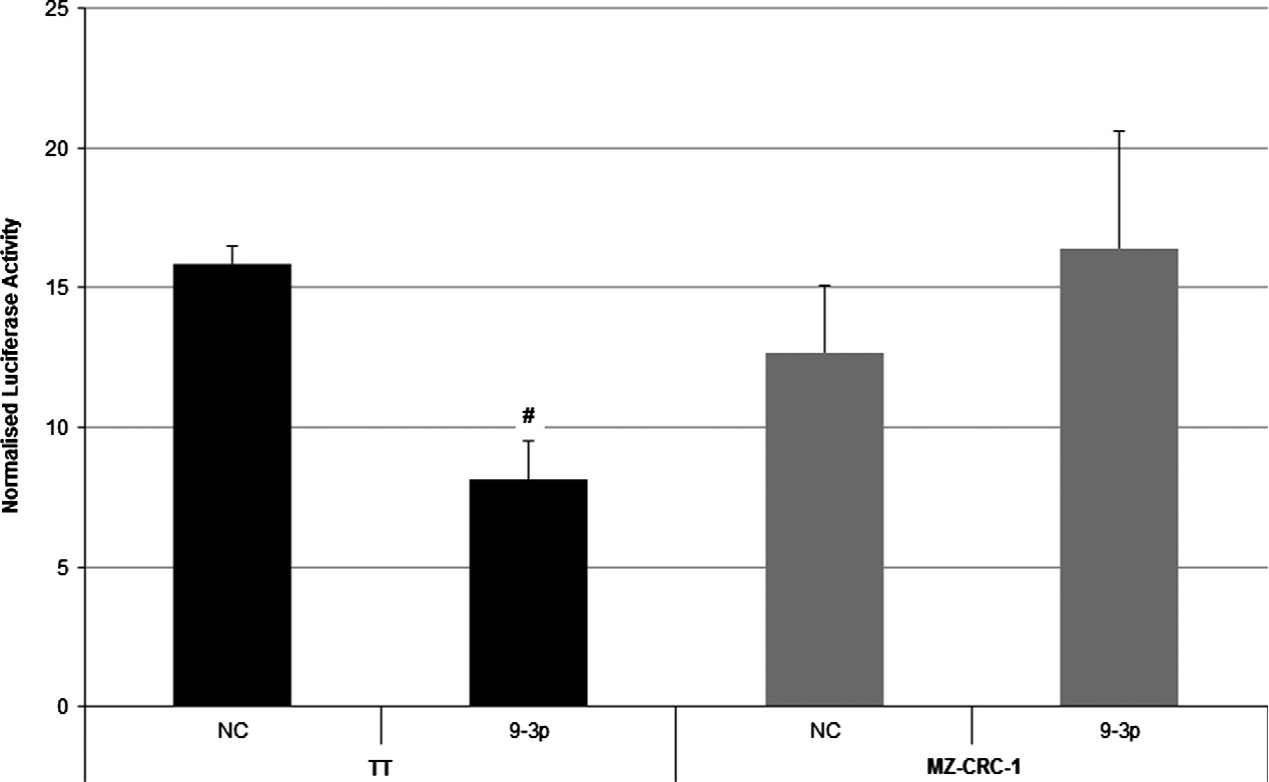
Normalized luciferase reporter assay results of TT and MZ-CRC-1 cells 48 h following miR-9-3p transfection (#*P *<* *0.05).

In order to test the hypothesis of miR-9-3p-targeted suppression of Atg5, a luciferase reporter assay was constructed and performed in both cell lines. This involved transient vector transfection and measurement of firefly and renilla activity. Figure4 shows a significant reduction in reporter assay signaling, following miR-9-3p transfection of the TT cell line at 48 h (*P *<* *0.01), consistent with targeted Atg5 inhibition. This effect was not seen, however, in the MZ-CRC-1 cell line, where subtle differences between negative control and miR-9-3p transfection were not significant (*P *=* *0.23).

### Atg5 siRNA knockdown inhibits cell proliferation and induces apoptosis

Additional Atg5 siRNA knockdown resulted in significantly reduced Atg5 protein expression in association with markedly elevated cleaved PARP, but only in the TT cell line (results not shown). Atg5 knockdown also led to reduced cellular proliferation, but not to the same extent as seen following miR-9-3p transfection with an observed reduction in the number of viable cells by 12.6% (TT cells; *P *=* *0.04) and 2% (MZ-CRC-1 cells; *P *=* *0.43), respectively. In keeping with the lack of elevated cPARP expression in the MZ-CRC-1 cell line, Atg5 knockdown did not influence cellular proliferation when compared to negative control transfection.

### Pharmacologic autophagy manipulation alters miR-9-3p expression

**Figure 5:**
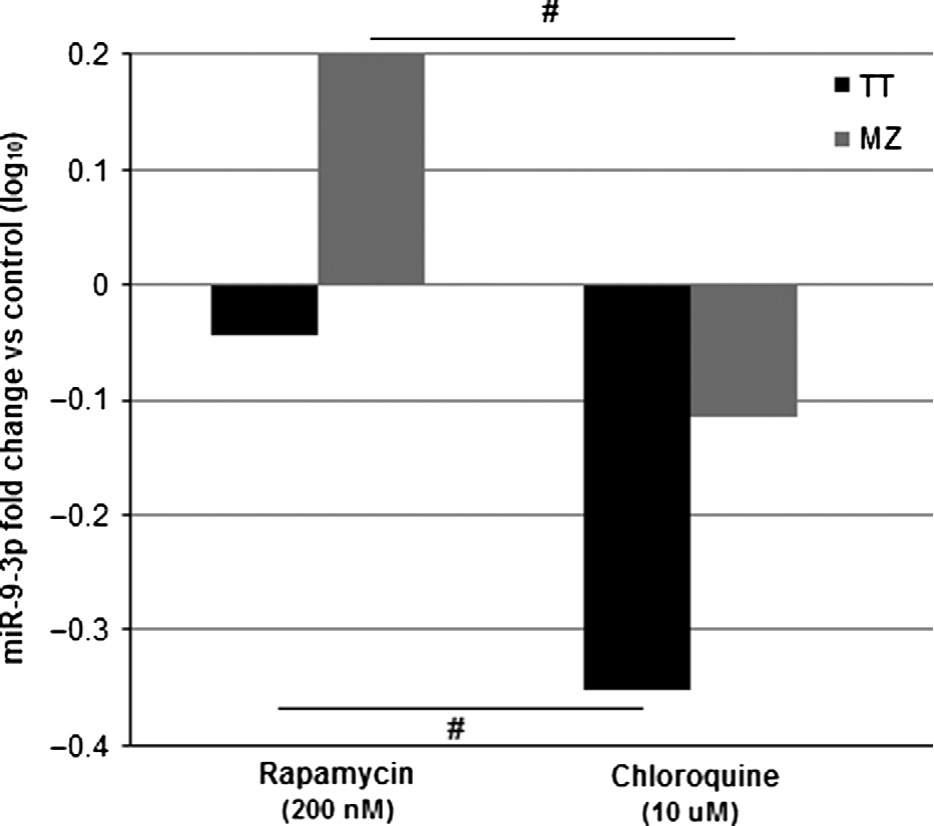
qPCR results displaying TT and MZ-CRC-1 miR-9-3p expression 48 h following treatment with rapamycin or chloroquine (expressed as log_10_ fold change relative to control; #*P *<* *0.05).

To further investigate the role of miR-9-3p regulation of autophagy, cell lines were treated with an autophagy inducer (Rapamycin) or a known autophagy blocker (Chloroquine) for 48 h. Using qPCR, significant differences were observed in miR-9-3p expression between treatments in both cell lines. More specifically, miR-9-3p expression was significantly higher after treatment with Rapamycin when compared to Chloroquine (Fig.5; TT: *P *=* *0.013; MZ-CRC-1: *P *<* *0.01).

This implies that miR-9-3p plays a regulatory role in autophagic flux in vitro*,* by attempting to maintain a basal level of autophagic flux; with expression elevating in circumstances of Rapamycin induced autophagy stimulation and expression being suppressed when autophagy is blocked with Chloroquine.

### Autophagic flux is increased in SMTC

Differences between hereditary (*n* = 6) and sporadic (*n* = 6) cases of MTC were investigated using a customized TLDA gene array card (as previously described for in vitro studies). Table S3 details significantly elevated autophagy genes (at *P *<* *0.1).

**Figure 6:**
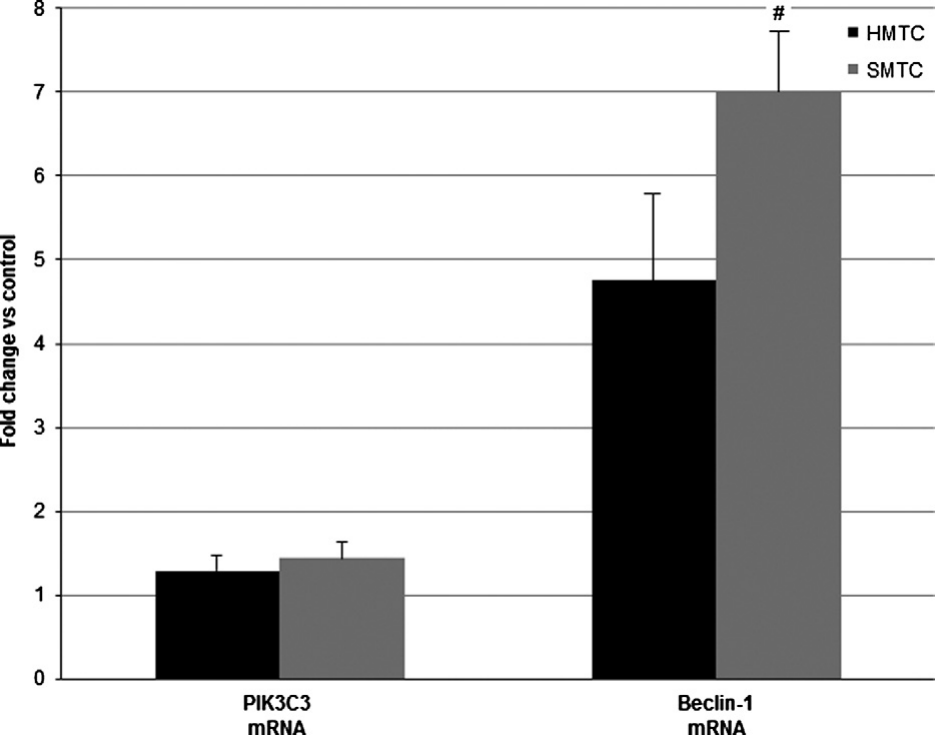
qPCR results displaying SMTC primary tumor expression of PIK3C3 and Beclin-1 as compared with HMTC (expressed as log_10_ fold change relative to HMTC; #*P *<* *0.05).

Validation studies involved comparison of hereditary (*n* = 8) and sporadic (*n* = 18) cases of disease with the genes of interest being *PIK3C3* (on the basis of the autophagy array data) and *Beclin-1*, which was selected given previously demonstrated importance in other cancer types. Selected autophagy gene mRNA expression results are presented in Figure6 and show that differential expression of *PIK3C3* mRNA expression was not supported by validation experiments (*P *=* *0.8). *Beclin-1* expression, however, was shown to be elevated in both HMTC and SMTC (when compared to normal thyroid control), with sporadic cases of disease possessing significantly higher expression when compared to hereditary (SMTC: 7 ± 0.8 vs. HMTC: 4.75 ± 1.0; *P *=* *0.042).

### Beclin-1 is a prognostic marker in SMTC

Following quantification of *Beclin-1* expression in the previously defined cohort of MTC cases, clinical data collation was undertaken and complete datasets were available in 19 cases (Table S4).

The median age of patients at diagnosis was 55 years and 58% were female. Thyroid nodule investigation was the most common presentation (79%), although two patients were identified following familial *RET* mutation screening and were ultimately found to have a diagnosis of MTC. A total thyroidectomy and bilateral central neck dissection was performed in all but one case at the index resection. An additional lateral neck dissection, ipsilateral and/or contralateral to the primary tumor was undertaken at the index resection where clinically indicated. Pathological assessment revealed a mean tumor size of 29 mm (±4). Fifty-three percent and 47% of patients were found to have positive central and lateral compartment nodal metastases, respectively, at the index resection. Five patients were found to be positive for germline *RET* gene mutations consistent with a diagnosis of HMTC.

Over a median follow-up time of 59 months, 79% went on to suffer from residual disease (i.e., persistent or recurrent). Distant metastases were diagnosed in 47% and mortality was observed in 26%.

Table S5 details *Beclin-1* expression correlations with a variety of clinicopathological outcome indices. Associations between *Beclin-1* expression and tumor size, extra-thyroidal extension or nodal metastases at diagnosis were not proven. However, with regard to clinical outcome, elevated *Beclin-1* expression was significantly associated with the development of residual disease.

## Discussion

MTC is a disease that has long been defined by the *RET* gene mutation; a discovery that has primarily assisted those with hereditary disease. There remains a need to further interrogate alternative strategies and novel mechanisms, particularly for those with sporadic disease. These studies have revealed further insights into the role of miRNA regulation of autophagy within the context of sporadic disease in particular.

Following prior studies 7, we demonstrated that transfection facilitated upregulation of miR-9-3p, results in therapeutic cell death in vitro. This was proven in two different (and presently the only well characterized) human MTC cell lines. This effect appeared more significant in TT cells as compared to the MZ-CRC-1 cell line.

Differential findings between cell lines were a common theme throughout experimentation. This may reflect the origins and molecular characteristics of these two cell lines which specifically harbor different *RET* gene mutations. It has recently been suggested that intratumoral heterogeneity may be a large factor in tumor adaptation and therapeutic response 13. It is likely that these issues are only magnified between cell lines from vastly different origins.

In keeping with this, a possible explanation for the greater therapeutic effect in the TT cell line relates to basal cell line miRNA expression. Preliminary findings (not shown in Results) showed that basal miR-9-3p expression was significantly higher in the MZ-CRC-1 cell line. While still engaging a therapeutic effect, it would appear that additional elevation of miR-9-3p in the cell line had a finite influence, and represented a relatively lower level of increased expression (compared to basal levels). In contrast, the low miR-9-3p expression seen in the TT cell line resulted in a greater relative change in miRNA expression following transfection that was subsequently associated with a larger therapeutic effect. This suggestion highlights the potential kinetics of miRNA regulation, in that changes in relative expression, rather than absolute expression, are a key regulatory determinant.

The mechanism of the miR-9-3p effect was investigated initially with cell cycle analysis. While significant G2 arrest was observed in the TT cell line, the change was relatively subtle, and this effect was absent in the MZ-CRC-1 cell line. Additionally, populations of sub-G1 cells, in either cell line, were not significantly altered by miR-9-3p transfection. Typically, a therapeutic effect (and particularly with G2 arrest) is accompanied by a large proportion of cells in apoptosis 14, which may manifest as an increased sub-G1 population of cells. This finding may relate to experimental methodology, whereby cells were washed twice prior to staining which may have reduced the number of necrotic and nonviable, apoptotic cells available for detection.

This is of interest given subsequent results delineating cleaved PARP immunoblotting expression. In both cell lines, following miR-9-3p transfection, cPARP expression was significantly elevated, providing more specific evidence of cell death via apoptosis. It could be assumed that miR-9-3p was enacting a mechanism based on cell death via apoptosis. However, these results were observed in parallel with suppression of markers of autophagy in both cell lines.

This raises a number of issues. Firstly, these findings are novel in identification of miR-9-3p regulation of autophagic flux in vitro*,* and in MTC specifically. Our previous studies had identified a suggestive change in autophagic flux following knockdown of miR-183 in the TT cell line 7; however, it was unclear on the basis of expression of just one marker of autophagy (LC3B) if this represented autophagy inhibition or activation. The current work, using a number of autophagy markers, demonstrated global suppression of autophagic flux following miR-9-3p transfection, in association with a therapeutic in vitro effect. The notion of miRNA regulation of autophagy is an evolving topic that has generated significant interest 10,15,16.

Secondly, the targeted influence of miR-9-3p was able to be isolated. More specifically, the only predicted target of miR-9-3p within the context of autophagy, Atg5, was shown through luciferase reporter experimentation to be a validated target (in the TT cell line). This has assisted in unlocking the precise mechanism of the miR-9-3p effect and identified an additional level of regulation that could be exploited therapeutically.

Employing targeted siRNA Atg5 knockdown as an adjuvant therapeutic strategy has been explored once previously in MTC. Lin et al. examined the in vitro impact of Atg5 suppression in combination with tyrosine kinase inhibitor (TKI) therapy and found that autophagy inhibition appeared to abrogate the therapeutic effect of TKI treatment 17. These results are in contrast to those of this study and the existing literature investigating the use of autophagy inhibition as a therapeutic strategy in parallel with existing treatment; as demonstrated in prostate cancer 18, chronic myeloid leukemia 19, hepatocellular carcinoma 20, gastrointestinal stromal tumors 21, and non-small-cell lung cancer 22. The findings of Lin and colleagues require additional validation.

Thirdly, experiments employing miR-9-3p and then also siRNA to Atg5 itself revealed not only autophagy inhibition, but evidence of crosstalk between autophagy and apoptosis pathways. This concept has been promoted by Xu et al., specifically within the context of miRNA 16, as well as Shen and colleagues, who suggested that the concept of cell death by autophagy is a misnomer 23. This is based on the assertion that cell death via autophagy does not exist, with any cell death within the context of autophagy being said to occur via alternative mechanisms; apoptosis for instance. The present experiments support this hypothesis, in that miR-9-3p transfection led not only to cell death and autophagy inhibition but also evidence of increased apoptotic flux. This suggests that autophagy pathway blockade was met by shunting of cellular processes through apoptosis.

Further to miR-9-3p autophagy inhibition and crosstalk with apoptosis, array studies were confirmatory of this influence throughout a multitude of autophagy-related genes. Additional validation studies revealed *BCL-2* and *LAMP-1* to be significantly suppressed following miR-9-3p transfection, reinforcing the suggestion of miRNA-induced autophagy inhibition and also revealing potential key regulators. Validation of *BCL-2* suppression is especially significant, given recent studies identifying antitumor activity through *BCL-2* downregulated induction of apoptosis 14.

The importance of *BCL-2* to regulation of apoptosis and the complex interplay with autophagy has been recognized previously 16,24, and a therapeutic role in this context has been identified in ovarian cancer 14. Rubenstein and Kimchi have gone on to suggest that *BCL2* may be the “single protein regulator” capable of regulating autophagy and apoptosis independently 24.

With regard to “autophagamiRs” 15, or miRNAs that play a role in autophagy regulation, pharmacologic induction and blockade experiments revealed the importance of miR-9-3p. Relatively elevated miR-9-3p expression was observed following autophagy induction, implying that miR-9-3p does maintain a regulatory influence in vitro. This could be explained as being akin to a negative feedback loop, whereby induction of a physiological process (in this case autophagy) is met with elevation of regulators (i.e., miR-9-3p) that reduce activity back to basal levels.

Lastly, when comparing clinical sporadic MTC tumors with hereditary cases, array studies revealed an almost universal elevation in autophagy-related genes in SMTC. Validation studies confirmed significantly elevated *Beclin-1* expression in SMTC. Additionally, expression correlated with clinical outcome; elevated expression identifying patients who developed residual disease.

Prognostic associations with *Beclin-1* have yielded conflicting results in a number of different cancer types 25–29. For instance, primary tumor overexpression has correlated with worse overall survival in colorectal cancer patients 25, whereas it has been shown to be a positive prognostic variable in esophageal squamous cell carcinoma 30, cervical cancer 27, and gastric cancer 26. Conflicting results may relate to methodology. In general, studies examining differential *Beclin-1* expression have compared cancer tissue with normal control tissue, as opposed to comparing two different groups within the same pool of cancers. It may be that MTC *Beclin-1* expression is relatively underexpressed, in general, when compared to normal parafollicular C cells. The absence of “normal” parafollicular control tissue has been an ongoing issue in MTC research.

Elevated Beclin-1 expression in sporadic tumors is suggestive of elevated basal autophagic flux. While there is no specific evidence presented here, it could be argued that autophagy is a tumor survival strategy, being engaged in times of cellular stress and ultimately resulting in a more aggressive disease phenotype. This is justified partly by this study, given in vitro evidence of autophagy inhibition resulting in cellular demise and additional primary tumor autophagy gene expression correlations with a worse clinical outcome. The concept of inducing autophagy inhibition in an adjuvant setting is gaining momentum and has recently been promoted in colon cancer 25, small-cell lung cancer 31, breast cancer 32, and melanoma 33.

Little previous work has been undertaken with regard to *Beclin-1*, thyroid cancer, and MTC specifically. One recent study, however, has examined functional overexpression of *Beclin-1* in thyroid cancer cell lines 34. Zhang and colleagues demonstrated that in vitro overexpression of *Beclin-1* sensitizes cells to treatment with proteasome inhibitors. Blockade of the ubiquitin–proteasome pathway would obviously be of future interest in treatment of sporadic MTC and is currently being employed clinically in refractory myeloma 35.

The known association of *Beclin-1* with *BCL2* is also of interest and while *Beclin-1* expression was examined in this study, it may be that its binding partner *BCL2* may be the master regulator of this relationship 24. Crosstalk between autophagy and apoptosis pathways is largely dependent on regulators such as *BCL2* and this could be the future research focus.

In conclusion, autophagy is a tumor cell survival mechanism in MTC. Disabling of autophagy is of additional therapeutic advantage through crosstalk with apoptosis. *Beclin-1* expression may be a useful biomarker of development of residual disease and tumor overexpression has revealed an additional therapeutic option with the use of proteasome inhibitor therapy.

## Conflict of Interest

None declared.

## Supporting Information

**Figure S1.** (A) TT cell cycle progression following miR-9-3p transfection versus negative control. (B) MZ-CRC-1 cell cycle progression following miR-9-3p transfection versus negative control (#*P *<* *0.05).

**Figure S2.** (A) western blot of apoptosis and autophagy markers following TT cell treatment with 3-MA or rapamycin; (B) quantified densitometry results of 5a (#*P *<* *0.05).

**Table S1.** (1) Pre-miR and siRNA assay identification numbers. (2) Western primary blot antibody identification numbers. (3) TLDA mRNA Autophagy Gene Targets. (4) qPCR probe identification numbers.

**Table S2.** Post-miR-9-3p transfection autophagy gene array results.

**Table S3.** Sporadic versus Hereditary MTC autophagy gene array results.

**Table S4.** Clinicopathological and outcome data for all study patients**.**

**Table S5.** Clinicopathological variables and associations with Beclin-1 expression.
