# Supplementary figures and images for: Noncoding RNA blockade of autophagy is therapeutic in medullary thyroid cancer
Source: Cancer Med. 2014 Dec 8;4(2):174–82. doi: 10.1002/cam4.355 (PMC4329002; doi:10.1002/cam4.355)

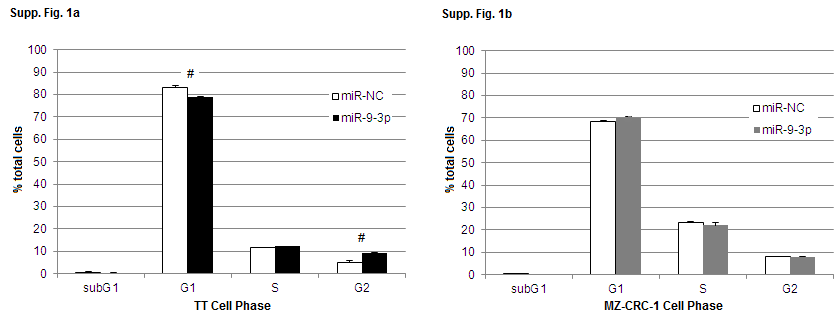

Supplement: Supplementary file 1 [file cam40004-0174-sd1.tif]

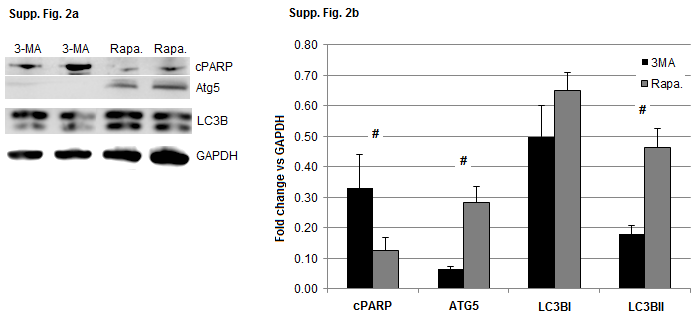

Supplement: Supplementary file 2 [file cam40004-0174-sd2.tif]
