# Supplementary material for: Noncoding RNA blockade of autophagy is therapeutic in medullary thyroid cancer
Source: Cancer Med. 2014 Dec 8;4(2):174–82. doi: 10.1002/cam4.355 (PMC4329002; doi:10.1002/cam4.355)
Supplement: Supplementary file 3 [file cam40004-0174-sd3.docx]

**Table S1.1:** pre-miR and siRNA assay identification numbers.

| **Pre-miR** | **ID** | **Source** |
| --- | --- | --- |
| Pre-miR-9-3p | #13072 | Applied Biosystems |
| Pre-miR-NC | #2118 | Applied Biosystems |
|  | | |
| **siRNA** | **ID** | **Source** |
| siATG5 | #2630 | Qiagen |
| siATG7 | #5114 | Qiagen |
| siBeclin-1 | #5114 | Qiagen |

| **Antibody** | **ID** | **Source** | **MW (kDa)** | **Dilution** |
| --- | --- | --- | --- | --- |
| β-Actin | A5441 | Sigma-Aldrich | 42 | 1:1000 |
| GAPDH | #2118 | Cell Signalling | 37 | 1:1000 |
| LC3B | #4108 | Cell Signalling | 14,16 | 1:1000 |
| Belcin-1 | #3738 | Cell Signalling | 60 | 1:1000 |
| ATG5 | #2630 | Cell Signalling | 55 | 1:1000 |
| p62 | #5114 | Cell Signalling | 60 | 1:1000 |
| cPARP | #9532 | Cell Signalling | 116 | 1:1000 |

**Table S1.2:** western primary blot antibody identification numbers.

**Table S1.3:** TLDA mRNA Autophagy Gene Targets.

| **Gene Symbols** | **1** | **2** | **3** | **4** | **5** | **6** | **7** | **8** | **9** | **10** | **11** | **12** |
| --- | --- | --- | --- | --- | --- | --- | --- | --- | --- | --- | --- | --- |
| **1** | AKT1 | AMBRA1 | APP | ATG10 | ATG12 | ATG16L1 | ATG16L2 | ATG3 | ATG4A | ATG4B | 18S | ATG4C |
| **2** | BNIP3 | CASP3 | CASP8 | CDKN1B | CDKN2A | CLN3 | CTSB | CTSD | CTSS | DAPK1 | DRAM1 | DRAM2 |
| **3** | HGS | HSP90AA1 | HTT | IFNG | IGF1 | INS | IRGM | LAMP1 | MAP1LC3A | MAP1LC3B | MAPK14 | MAPK8 |
| **4** | SNCA | TGFB1 | TGM2 | TMEM74 | TNF | TNFSF10 | TP53 | ULK1 | ULK2 | UVRAG | WIPI1 | ACTB |
| **1** | ATG4D | ATG5 | ATG7 | ATG9A | ATG9B | BAD | BAK1 | BAX | BCL2 | BCL2L1 | BECN1 | BID |
| **2** | CXCR4 | EIF2AK3 | EIF4G1 | ESR1 | FADD | FAS | GAA | GABARAP | GABARAPL1 | GABARAPL2 | HDAC1 | HDAC6 |
| **3** | MTOR | NFKB1 | NPC1 | PIK3C3 | PIK3CG | PIK3R4 | PRKAA1 | PTEN | RAB24 | RB1 | RGS19 | RPS6KB1 |
| **4** | B2M | GAPDH | HPRT1 | PSMC4 | CDKN1A | IPO8 | GUSB | RPLP0 | BCL2 | CASP3 | CASP8 | MTOR |

**Table S1.4:** qPCR probe identification numbers.

| **mRNA** | **ID** | **Source** |
| --- | --- | --- |
| GAPDH | #Hs02758991_g1 | Applied Biosystems |
| β-Actin | #Hs01060665_g1 | Applied Biosystems |
| Beclin-1 | #Hs00186838_m1 | Applied Biosystems |
| ATG5 | #Hs00169468_m1 | Applied Biosystems |
| PIK3C3 | #Hs00176908_m1 | Applied Biosystems |
| mTOR | #Hs00234508_m1 | Applied Biosystems |
| LAMP-1 | #Hs00174766_m1 | Applied Biosystems |
| PCDH10 | ##Hs00252974_s1 | Applied Biosystems |
|  | | |
| **miRNA** | **ID** | **Source** |
| hsa-miR-9-3p | #002231 | Applied Biosystems |
| hsa-miR-183 | #002269 | Applied Biosystems |
| hsa-miR-375 | #000564 | Applied Biosystems |
| RNU48 | #001006 | Applied Biosystems |
