# Supplementary material for: Noncoding RNA blockade of autophagy is therapeutic in medullary thyroid cancer
Source: Cancer Med. 2014 Dec 8;4(2):174–82. doi: 10.1002/cam4.355 (PMC4329002; doi:10.1002/cam4.355)
Supplement: Supplementary file 4 [file cam40004-0174-sd4.doc]

**Supp. Table 2:** Post miR-9-3p transfection autophagy gene array results.

| **TT** | **Fold Change** | ***P*** |
| --- | --- | --- |
| MTOR-Hs00234522_m1 | 0.4477 | 0.0002 |
| WIPI1-Hs00215872_m1 | 0.5302 | 0.0519 |
| UVRAG-Hs00163433_m1 | 0.6008 | 0.0683 |
| PTEN-Hs02621230_s1 | 0.6587 | 0.0957 |
| **MZ-CRC-1** |  | |
| BCL2-Hs00153350_m1 | 0.5574 | 0.0046 |
| TGM2-Hs00190278_m1 | 0.3395 | 0.0046 |
| ULK1-Hs00177504_m1 | 0.4403 | 0.0095 |
| APP-Hs00169098_m1 | 0.473 | 0.0135 |
| RPLP0-Hs99999902_m1 | 0.8189 | 0.0183 |
| LAMP1-Hs00174766_m1 | 0.4516 | 0.0208 |
