# Supplementary material for: Noncoding RNA blockade of autophagy is therapeutic in medullary thyroid cancer
Source: Cancer Med. 2014 Dec 8;4(2):174–82. doi: 10.1002/cam4.355 (PMC4329002; doi:10.1002/cam4.355)
Supplement: Supplementary file 5 [file cam40004-0174-sd5.doc]

**Supp. Table 3:** Sporadic vs Hereditary MTC autophagy gene array results.

| **SMTC (vs HMTC)** | **Fold Change** | ***P*** |
| --- | --- | --- |
| PIK3C3-Hs00176908_m1 | 2.451 | 0.0083 |
| IPO8-Hs00183533_m1 | 2.5015 | 0.0232 |
| RPS6KB1-Hs00177357_m1 | 2.4568 | 0.0396 |
| PRKAA1-Hs01562315_m1 | 2.8265 | 0.0431 |
| WIPI1-Hs00215872_m1 | 2.1086 | 0.0557 |
| HTT-Hs00918174_m1 | 4.1654 | 0.0612 |
| MAP1LC3B-Hs00797944_s1 | 2.9847 | 0.0989 |
