# Supplementary material for: Noncoding RNA blockade of autophagy is therapeutic in medullary thyroid cancer
Source: Cancer Med. 2014 Dec 8;4(2):174–82. doi: 10.1002/cam4.355 (PMC4329002; doi:10.1002/cam4.355)
Supplement: Supplementary file 6 [file cam40004-0174-sd6.docx]

**Supp. Table 4:** Clinicopathological and outcome data for all study patients**.**

| Patient | Age at diagnosis | Sex | Tumour size (mm) | Extra-thyroidal extension | Lympho-vascular invasion | Germline | *RET* gene analysis | Operative Code | Lateral nodal metastases | Follow up (months) | Residual disease | Distant metastases | Mortality (months post op.) |
| --- | --- | --- | --- | --- | --- | --- | --- | --- | --- | --- | --- | --- | --- |
| 1 | 34 | M | 55 |  |  | S |  | 3 |  | 141 | N |  | NA (alive) |
| 2 | 60 | F | 8 |  |  | S |  | 2 | Y | 140 | persistent | Y | 140 |
| 3 | 22 | F | 55 |  |  | S |  | 3 |  | 220 | persistent |  | NA (alive) |
| 4 | 55 | F | 18 |  | Y | S |  | 2 | Y | 59 | persistent | Y | NA (alive) |
| 5 | 77 | F | 20 |  | Y | S |  | 3 |  | 115 | N |  | 115* |
| 6 | 22 | F | 24 | Y | Y | S |  | 2 | Y | 50 | persistent |  | NA (alive) |
| 7 | 62 | M | 20 |  | Y | S |  | 2 | Y | 39 | persistent | Y | 39 |
| 8 | 64 | M | 30 |  |  | S |  | 2 | Y | 18 | persistent | Y | 18 |
| 9 | 65 | M | 60 |  |  | S |  | 2 | Y | 197 | recurrent | Y | NA (alive) |
| 10 | 20 | F | 23 |  |  | H | C634Y | 1 |  | 36 | N |  | NA (alive) |
| 11 | 24 | M | 55 |  |  | H | 918 | 2 |  | 51 | persistent | Y | NA (alive) |
| 12 | 54 | M | 20 | Y | Y | H | C618R | 2 | Y | 175 | recurrent |  | NA (alive) |
| 13 | 27 | F | 15 |  |  | H | C618R | 2 | Y | 194 | persistent | Y | NA (alive) |
| 14 | 62 | F | 12 |  | Y | H | 620 | 3 |  | 128 | persistent |  | NA (alive) |
| 15 | 54 | F | 21 | not rep. | not rep. | S |  | 2 |  | 65 | persistent |  | NA (alive) |
| 16 | 63 | M | 54 | Y | Y | S |  | 2 | Y | 20 | persistent | Y | 20 |
| 17 | 39 | F | 14 |  |  | S |  | 3 |  | 22 | N |  | NA (alive) |
| 18 | 76 | M | 25 | Y | Y | S |  | 2 | Y | 22 | persistent | Y | NA (alive) |
| 19 | 55 | F | 17 |  | Y | S |  | 2 | Y | 59 | persistent |  | NA (alive) |

**Key:** F: female; H: hereditary; M: male; S: sporadic; Y: yes; F/Up: follow up; op.: operative; operative code: 1: total thyroidectomy; 2: total thyroidectomy, bilateral central neck dissection and ipsilateral lateral neck dissection; 3: 2 and “other” lymph node dissection; NA: not applicable; *: denotes mortality due to comorbidity (not due to disease of interest).
