# Supplementary material for: Noncoding RNA blockade of autophagy is therapeutic in medullary thyroid cancer
Source: Cancer Med. 2014 Dec 8;4(2):174–82. doi: 10.1002/cam4.355 (PMC4329002; doi:10.1002/cam4.355)
Supplement: Supplementary file 7 [file cam40004-0174-sd7.docx]

**Supp. Table 5:** Clinicopathological variables and associations with Beclin-1 expression.

| **Variable** |  |  | **Beclin-1 (OR; 95%CI)** | ***P*** |
| --- | --- | --- | --- | --- |
| **N** | SMTC | 14 (74%) |  |  |
|  | HMTC | 5 (26%) |  |  |
| **Age** |  | median: 55  (range: 20-77) |  | 0.42 |
| **Gender** | M | 8 (42%) |  | 0.32 |
|  | F | 11 (58%) |  |  |
| **Tumour size (mm)** | (mean±SE) | 29 ± 4.0 | 1.07(-1.5-3.65) | 0.39 |
| **Extra-thyroidal extension** |  | 5 (27%) | 0.51(0.22-1.18) | 0.12 |
| **Positive central nodes** |  | 10 (53%) | 0.91(0.69-1.21) | 0.53 |
| **Positive lateral nodes** |  | 9 (47%) | 0.94(0.71-1.23) | 0.64 |
| **Residual disease** |  | 15 (79%) | 1.3(1.11-1.9) | **0.04** |
| **Distant metastases** |  | 9 (47%) | 0.91(0.68-1.21) | 0.50 |
| **Mortality** |  | 5 (26%) | 0.98(0.74-1.34) | 0.98 |

(***key*:** CI: confidence interval; F:female; HMTC: hereditary MTC; OR: odds ratio; SMTC: sporadic MTC; M:male; SE: standard error)
