# Supplementary material for: Noncoding RNA blockade of autophagy is therapeutic in medullary thyroid cancer
Source: Cancer Med. 2014 Dec 8;4(2):174–82. doi: 10.1002/cam4.355 (PMC4329002; doi:10.1002/cam4.355)
Supplement: Supplementary file 8 [file cam40004-0174-sd8.docx]

***Cell culture***

The TT and MZ-CRC-1 human MTC cell lines were employed for all *in vitro* experimentation (characterised for authenticity by CellBank Australia). TT cells were cultured in HyClone® F-12 Kaighn’s Modification medium (F-12K; HyClone Laboratories, Thermo-Scientific) and MZ-CRC-1 cells in HyClone® DMEM medium (DMEM; HyClone Laboratories, Thermo-Scientific); both being supplemented with 10% fetal calf serum. Cells were cultured in a humidified chamber (5%CO_2_, 37°C) and were confirmed mycoplasma free.

***Transfection of Pre-miR or siRNA***

The experimental transfection protocol involved reverse pre-miR or siRNA transfection (using lipofectamine® RNAiMax, Invitrogen) at a concentration of 50nM. The majority of experimentation involved a 48hour post reverse transfection end-point given previous success in our laboratory with this time point.[^7^](#_ENREF_7) The specifics of pre-miRs and siRNAs used in these studies are available in supplementary data (Table S1.1).

***Drug treatment***

Rapamycin (Sigma-Aldrich, #8781) was obtained as a ready-made 2.74mM solution and experiments were performed over a range of concentrations (5-400nM). Chloroquine diphosphate salt, lyophilised powder (Sigma-Aldrich, #C6698) was re-suspended before experimentation over a range of concentrations (5-40µM). 3-methlyadenine (3MA; Sigma-Aldrich, #M9281) was also obtained as a lyophilised powder and was re-suspended before use (concentration range: 1-10µM).

***Cell proliferation assay***

Experiments were undertaken in 96-well plates, in which 1.0-1.5x10^4^ cells per well were seeded. The CellTiter 96^®^ AQ_ueous_ One Solution Cell Proliferation Assay (MTS assay; Promega) was employed throughout (absorbance measured at 490nm using a 96-well plate reader; Sunrise™ Microplate Reader; Tecan).

***Clonogenic assay***

Reverse transfected cells were lifted and 2000 cells per well were re-plated into 6-well plates. Culture medium was changed every ten days and at 28d, cells were washed and stained (0.25g methylene blue power, 50ml 100% ethanol and 50ml distilled water). Cell colonies were captured using an LAS4000 digital imaging system (Fujifilm, Tokyo, Japan) and quantified with Colony software (V1.1; Fujifilm).

***Cell cycle analysis***

The pre-miR effect upon cell cycle progression was assessed using propidium iodide (PI) staining and fluorescence-activated cell sorting (FACS) analysis (FACS Calibre; BD Biosciences). Following experimental treatment, cells were washed with PBS, lifted and re-suspended with PBS in a glass FACS tube. To this, 200µl 5%triton X-100, 50µl of RNaseA (Qiagen) and 200µl of PI were added and mixed by pipetting. The stained cell suspension was vortexed briefly and placed on the FACS Calibre aspiration mount before CellQuest Pro™ software (BD Biosciences) acquired data. Results were quantified with Modfit™ software (V3.2; Verity Software House).

***Luciferase reporter assay***

Experiments were geared toward specific targeting of Atg5 by miR-9-3p given

prediction modeling software identification of this putative target based on 3’UTR base pair complementarity (http://pictar.mdc-berlin.de/). The firefly luciferase gene (*luc2*) was employed as the primary reporter, which was cloned with the miRNA target sequence and inserted via vector transfection into cell lines (achieved with pmirGLO Dual-Luciferase® miRNA Target Expression Vector; Promega). The reporter assay signal was quantified with the Dual-Luciferase® Reporter Assay System (DLR™ Promega). Reverse co-transfected cell lysates were pipetted into a 96-well plate in triplicate and loaded into a luminometer (Veritas™ Microplate Luminometer, Turner BioSystems). Luciferase activity was quantified by calculating the firefly to *Renilla* reporter assay signal ratio.

***Western blot analysis***

20µg of denatured total protein was separated using a precast gel (4-12%; Invitrogen). The membrane was blocked overnight at 4^o^C in either 5% skim milk powder/TBST (Tris buffered saline solution with Tween-20: 150nM NaCl, 10mM Tris HCi, 1% Tween-20) or 5% bovine serum albumin/TBST (BSA/TBST). After blocking, the membrane was incubated for one hour at room temperature with primary antibody (in 5% skim milk powder/TBST or 5% BSA/TBST). Bound primary antibody was detected by washing the membrane in monoclonal goat anti-mouse horseradish peroxidase-conjugated secondary antibody (Dako) or polyclonal goat anti-rabbit horseradish peroxidase (Dako). This was followed by exposure to a horseradish peroxidase chemiluminescence substrate for 5min. (ECL Plus Western Blotting Detecting System, GE Healthcare). Resulting bands were captured using an LAS4000 digital imaging system (Fujifilm) and densitometry was quantified with MultiGauge software (V3.0; Fujifilm). Primary antibody details are available in supplementary files (Table S1.2).

***Primary tumour samples and clinical data***

Fresh frozen primary tumour tissue was obtained from the Kolling Institute of Medical Research, Neuroendocrine Tumour Bank. This tissue was used to undertake autophagy gene mRNA array profiling of sporadic versus hereditary MTC. Additional cases with available fresh frozen tumour bank tissue or archived formalin fixed paraffin embedded (FFPE) tissue were selected as the cohort for validation of array results. This tissue was sourced from the aforementioned tumour bank, archived tissue from the Royal North Shore Hospital (Sydney, Australia) or the Alfred Hospital (Melbourne, Australia) Departments of Anatomical Pathology. Clinical data for these patients was collated from the prospectively maintained University of Sydney, Endocrine Surgical Unit and Monash University, Alfred Endocrine Surgical Unit databases, medical records and pathology reports.

***Total RNA extraction***

Total RNA isolation was achieved using the miRNeasy^®^ Mini total RNA Isolation Kit (for cell lines, conditioned media, fresh frozen tumour tissue; Qiagen) or the miRNeasy^®^ FFPE Kit (for FFPE samples; Qiagen). Nucleic acid quantification was performed on the Nanodrop 1000^®^ spectrophotometer (Nanodrop Technologies).

***Autophagy gene array***

Autophagy gene mRNA array studies were performed on total RNA samples extracted from primary MTC tumours and cell lines (48h post pre-miR-9-3p transfection). Array studies were conducted on a customised TaqMan^®^ Low Density Array (TLDA) gene card (Applied Biosystems) which contained probes for 48 autophagy related genes (available in supplementary data; S1.3).

***qRT-PCR***

Cell line and primary tumour miRNA expression was quantified using TaqMan^®^

miRNA assays (details in Supplementary data, S1.4; Applied Biosystems) on the ABI 7900 HT Fast System (Applied Biosystems). Assays were completed in two-steps (first step: reverse transcription of total RNA to cDNA; second step: PCR amplification of cDNA using the 5’ nuclease technique and TaqMan^®^ 2x Universal PCR Master Mix No AmpErase^®^ UNG; Applied Biosystems). All qPCR was performed using the ABI7900 HT Fast System and cycled according to recommended conditions. Data analysis was performed using RQ Manager (V1.2.1; Applied Biosystems) and DataAssist (V3.01; Applied Biosystems) software. Relative expression was quantified using the ΔΔC_T_ method against an endogenous control gene (RNU48).

Selected mRNAs investigated for validation in cell lines and primary tumour samples. TaqMan^®^ assays (Applied Biosystems; S1.4) were used to detect and quantify mRNA expression and assays were also completed in two-steps (first step: reverse transcription of total RNA to cDNA using the High Capacity RNA-to-cDNA™ kit; Applied Biosystems; second step: PCR amplification of cDNA using the 5’ nuclease technique and TaqMan^®^ Gene Expression Master Mix; Applied Biosystems). Cycling and data analysis were performed as above, with use of GAPDH and β–actin as endogenous controls.

***Statistical analysis***

Nominal clinical outcome data was assessed with Fisher’s exact or Chi-Squared tests and analyses of non-parametric continuous variables were conducted with a Mann-Whitney U Test. Where approprite, independent T-tests were applied. Univariate and multivariate logistic regression was used to identify mRNA expression associations with outcome. Autophagy gene mRNA array data was analysed using a two-tailed t-test with post-hoc Benjamini-Hochberg false discovery correction (*P*<0.05). The statistical package Stata/IC12 (Version 12.1, StataCorp LP) was employed to perform analyses. *P*<0.05 was considered significant.
